# Pharmacokinetics of ascending doses of ivermectin in *Trichuris trichiura*-infected children aged 2–12 years

**DOI:** 10.1093/jac/dkz083

**Published:** 2019-03-11

**Authors:** Jessica D Schulz, Jean T Coulibaly, Christian Schindler, David Wimmersberger, Jennifer Keiser

**Affiliations:** 1 Department of Medical Parasitology and Infection Biology, Swiss Tropical and Public Health Institute, Basel, Switzerland; 2 University of Basel, Basel, Switzerland; 3 Unité de Formation et de Recherche Biosciences, Université Félix Houphouët–Boigny, Abidjan, Côte d’Ivoire; 4 Centre Suisse de Recherches Scientifiques en Côte d’Ivoire, Abidjan, Côte d’Ivoire

## Abstract

**Background:**

Yearly, millions of children are treated globally with ivermectin mainly for neglected tropical diseases. Anatomical, physiological and biochemical differences between children and adults may result in changes in pharmacokinetics. However, paediatric pharmacokinetic data of ivermectin are lacking.

**Methods:**

In the framework of a randomized controlled dose-finding trial in rural Côte d’Ivoire, *Trichuris trichiura*-infected pre-school-aged children (PSAC, 2–5 years) and school-aged children (SAC, 6–12 years) were assigned to 100 or 200 μg/kg and 200, 400 or 600 μg/kg ivermectin, respectively (ISRCTN registry no. ISRCTN15871729). Capillary blood was collected on dried blood spot cards until 72 h post-treatment. Ivermectin was quantified by LC-MS/MS, and pharmacokinetic parameters were evaluated by non-compartmental analysis.

**Results:**

*C*_max_ and AUC increased in PSAC and SAC with ascending doses and were similar in both age groups when the current standard dose (200 μg/kg) was administered (∼23 ng/mL and ∼350 ng×h/mL, respectively). PSAC with lower BMI were associated with significantly higher AUCs. AUC and *C*_max_ were ∼2-fold lower in children compared with parameters previously studied in adults, whereas body weight-adjusted CL/F (∼0.35 L/h/kg) was significantly higher in children. *T*_max_ (∼6 h), *t*_1/2_ (∼18 h), mean residence time (MRT_INF_) (∼28 h) and V/F (∼8 L/kg) were similar in all paediatric treatment arms.

**Conclusions:**

A positive association of AUC or *C*_max_ with dose was observed in both age groups. Undernutrition might influence the AUC of ivermectin in PSAC. Ivermectin shows a lower exposure profile in children compared with adults, highlighting the need to establish dosing recommendations for different age groups.

## Introduction

Ivermectin is an antiparasitic marketed to orally treat onchocerciasis and strongyloidiasis, and is used in the combination with albendazole against lymphatic filariasis.^1^ It is known for its broad antiparasitic activity and thus is currently being explored as an alternative treatment for rabies, mansonellosis, cancer and other diseases, as well as as a tool for malaria transmission control.^2–5^ Additionally, promising efficacy against soil-transmitted helminth (STH) infections was observed, and the combined therapy of ivermectin with the standard drug albendazole has therefore been recently added to the Essential Medicine List for this indication.^3^ STH infections belong to the group of neglected tropical diseases and are caused by infections with *Ascaris lumbricoides*, hookworms (*Necator americanus*, *Ancylostoma duodenale* and, to a smaller extent, *Ancylostoma ceylanicum*) and *Trichuris trichiura*. A total of 20% of the world’s population are estimated to be infected with at least one of the STHs, and the parasites are endemic in most countries of Central and South America, Africa and Asia.^6^ Annual or bi-annual large-scale treatment of populations at (high) risk, so-called targeted preventive chemotherapy, is the current strategy of the WHO to control the burden of soil-transmitted helminthiasis.^7–9^ However, none of the recommended drugs can effectively treat *T. trichiura.*^10^

Even though ivermectin has been in use since the early 1980s, it has not been systematically evaluated in human medicine (e.g. for safety, effective doses and drug disposition in different populations). To date, pharmacokinetic (PK) studies of ivermectin have been conducted mostly in a low number of healthy adults or adults infected with *Onchocerca volvulus* or *Plasmodium falciparum* malaria.^11–16^ Yet, a PK characterization is essential to understand the human body’s response to a drug, especially in populations that physiologically differ from healthy adults, such as children. Additionally, physiological abnormalities such as malnutrition or undernutrition and intestinal worms, which are common public health problems in developing countries, can potentially affect drug disposition.^17^ Since most parasitic diseases affect mainly children, the characterization of ivermectin’s PK in this age group is urgently needed to apply it safely and effectively to a broad range of diseases.

For the first time, a PK trial was conducted in rural Côte d’Ivoire with 120 school-aged children (SAC, 6–12 years) and 80 pre-school-aged children (PSAC, 2–5 years) infected with *T. trichiura* in the framework of a phase II dose-finding study. Children were treated with ascending doses of ivermectin, namely 100 or 200 μg/kg for PSAC and 200, 400 or 600 μg/kg for SAC. A micro-blood sampling technique was performed to collect dried blood spot (DBS) samples over 72 h. Ivermectin was extracted from the DBS samples and quantified with a previously validated LC-MS/MS method.^18^ PK parameters were evaluated and correlated to ivermectin’s efficacy against *T. trichiura* and to anthropological measures. Finally, PK parameters were compared between the two age groups and with ivermectin’s PK in adult volunteers, which were reported previously.^18^

## Materials and methods

### Chemicals and material

Ivermectin (powder, 96% B1A), formic acid (LC-MS grade) and ammonium acetate (LC-MS grade) were purchased from Sigma–Aldrich (Buchs, Switzerland). Ivermectin-d_2_ was synthesized by Toronto Research Chemicals (Ontario, Canada). Ivermectin tablets (3 mg) were kindly provided by ELEA (Buenos Aires, Argentina). Ivermectin mini-tablets at a strength of 500 μg were produced at the University of Basel.^19^ Ultrapure water was prepared using a Millipore water purification system (Milli-Q^®^ Advantage A10, Merck, Darmstadt, Germany). LC-MS-grade solvents, acetonitrile and isopropanol, and Whatman^®^ protein saver cards 903 were purchased from Merck KGaA (Darmstadt, Germany). SOLAμ solid phase extraction (SPE) plates HRP (hydrophilic reversed phased) were obtained from Thermo Fisher Scientific (Reinach, Switzerland) and protein low-binding 96-well plates (PCR clean) were purchased from Vaudaux-Eppendorf AG (Basel, Switzerland). 

### Study design, procedure and ethics considerations

The PK study was embedded in a phase II randomized, single-blind trial in rural Côte d’Ivoire with the primary objective of identifying the efficacy of ascending, single oral doses of ivermectin against *T. trichiura* infections. Additionally, the tolerability of the interventions was evaluated by clinical examinations, assessment of adverse events and blood analysis. Efficacy and safety data, as well as detailed information on inclusion and exclusion criteria, randomization procedure and diagnostic methods are published elsewhere.^19^ Ethics approval was obtained from the Ethical Committee of Northwestern and Central Switzerland (2017-00250) and the Comité d’Ethique et de la Recherche of the Ministry of Health in Côte d’Ivoire (052/fMSHP/CNER-kp). The study was registered at the ISRCTN registry (no. ISRCTN15871729). Volunteers were first invited to information events and, thereafter, written informed consent was obtained from parents or guardians of all children, and SAC gave verbal assent.

For the PK study, 80 PSAC (2–5 years of age) and 120 SAC (6–12 years of age) with *T. trichiura* infection (>60 eggs/g of stool for PSAC and >100 eggs/g of stool for SAC) were enrolled in the trial in the setting of Azaguié, Côte d’Ivoire. Prior to treatment, children were examined for anthropometric measures, i.e. weight and height. PSAC were randomly assigned to two treatment arms (100 or 200 μg/kg ivermectin) and SAC to three treatment groups (200, 400 or 600 μg/kg ivermectin). On the treatment day, participants received a standardized fatty breakfast (oily fish on bread) owing to ivermectin’s enhanced bioavailability following fatty food intake.^20^ Thereafter, ivermectin tablets were orally administered with a glass of water and treatment time was recorded. SAC received 3 mg tablets and PSAC 0.5 mg mini-tablets according to dose and weight. The study nurses performed micro-blood sampling at 0, 1, 2, 4, 6, 7, 8, 9, 24, 48 and 72 h post-treatment by taking capillary blood. Sterile finger-prickers were used to puncture the tip of a finger of the participants to obtain a drop of blood. Lithium heparin-coated capillaries were loaded with blood, which was subsequently dropped onto DBS cards (∼60 μL per spot). This was performed in four replicates for each patient and timepoint. The DBS cards were allowed to dry for at least 2 h and then stored at room temperature at the clinical trial site in sealed plastic bags containing silica desiccants. DBS samples were shipped to Basel, Switzerland and stored at −80°C until they were processed for analysis.

### Sample extraction and analysis by LC-MS/MS

The development, optimization and validation of ivermectin extraction from DBS samples and the analytical LC-MS/MS method are described elsewhere.^18^ When the DBS samples from participants were analysed, calibration line samples (3, 5, 10, 25, 50, 80, 100, 150 and 200 ng/mL ivermectin), quality control samples (six replicates of 3, 5, 100 and 180 ng/mL ivermectin) and blank samples (pure blood extracted from DBS) with internal standard (ivermectin-d_2_) were extracted and analysed simultaneously. US FDA guidelines require a linearity of the calibration line of *r^2^* >0.99 and an accuracy of 3/4 of calibration line and 2/3 of quality control samples of ±15% [±20% for the lower limit of quantification (LLOQ)] versus the nominal value.^21^

### Data analysis

PK parameters were obtained by non-compartmental analysis using WinNonlin (5.2, Certara, Princeton, NJ, USA). Maximum ivermectin concentrations (*C*_max_) and time to reach *C*_max_ (*T*_max_) were observed values. The half-life (the time in which half of the absorbed drug is eliminated) was calculated as *t*_1/2_=ln(2)/λ_Z_. AUC was determined until the last measurement (AUC_0–72_) and until infinity (AUC_INF_). The area under the first-moment curve was evaluated until infinity (AUMC_INF_). AUCs and AUMC_INF_ were calculated using the linear trapezoidal rule. The mean residence time (MRT_INF_) was determined by AUMC_INF_/AUC_INF_, and drug clearance (CL/F) was assessed by dose/AUC_INF_. CL/F was further adjusted to the participants’ weights. The apparent volume of distribution (V/F) was evaluated by (CL/F)/λ_Z_/kg.

Statistical analysis was performed with GraphPad Prism 6.01 (GraphPad, CA, USA) and Stata Statistical Software: Release 14 (StataCorp LLC, College Station, TX, USA). Kruskal–Wallis analysis followed by Dunn’s post-test was performed to compare PK parameters (*C*_max_, AUC_0–72_ and CL) between treatment arms or age groups. Significance (*P* value) is illustrated in the figures.

Additionally, a dose–response model of the following form was estimated:

$$y=b_{0}+{\;b}_{1}\times \;x\;+\;\left(b_{2}+{\;b}_{3}\times \;x\right)\;\times {\text{ dose}}^{\;\text{exp}(b4)},$$

where x=weight or BMI and y=AUC_INF_ or *C*_max_. Four specific models were compared using the Akaike information criterion (AIC): (a) b_3_, b_4_ ≠ 0; (b) b_3_ ≠ 0, b_4_=0; (c) b_3_=0, b_4_ ≠ 0; and (d) b_3_, b_4_=0. The model with the lowest AIC was plotted for different values of weight or BMI. Cure rates represent the percentage of volunteers who were fully cured (egg negative) after treatment. Egg reduction rates are defined by the group geometric mean reduction in the number of excreted eggs from baseline (prior to treatment) to follow-up (2–3 weeks post-treatment) diagnosis.^19^

## Results

### Study participants and micro-blood sampling

Participants’ characteristics are summarized in Table 1. In total, 120 SAC [receiving 200 μg/kg (*n* = 41), 400 μg/kg (*n* = 39) or 600 μg/kg (*n* = 40)] participated in the PK study, and a complete DBS sample set was available for the treatment day (0–9 h) and the 24 h timepoint, but two and four DBS samples at 48 h and 72 h, respectively, could not be collected. A total of 80 PSAC were enrolled and treated with 100 μg/kg (*n* = 39) or 200 μg/kg (*n* = 41) ivermectin. In total, nine DBS samples of PSAC were not taken on the day of treatment (1 × 2 h, 1 × 7 h, 3 × 8 h, 4 × 9 h), one DBS sample was missed at each of the 24 and 72 h timepoints and seven participants were not available for DBS sampling at 48 h.

**Table 1.** Participant characteristics

Parameter	PSAC *(n* = 80)	SAC (*n* = 120)
Female, *n* (%)	40 (50)	50 (42)
Age (years)	4 (2–5)	8 (6–12)
Height (cm)	93 (78–111)	121 (91–156)
Weight (kg)	15 (13–18)	22 (12–49)
BMI	16 (12–25)	15 (12–24)

Data are presented as mean (range) unless otherwise stated.

### LC-MS/MS analysis of DBS samples

The calibration line of all experiments fulfilled requirements with *r^2^* >0.994. A minimum of 3/4 of the calibration line and 2/3 of quality control samples passed accuracy with ±15% (±20% for LLOQ) versus the nominal value. The extraction and analysis of 7% of samples were repeated and 72% (>2/3) deviated <20% from the initial analysed concentrations. The LLOQ for DBS samples is 3 ng/mL. DBS samples that resulted in <3 ng/mL were set to 0 ng/mL.

### PK parameters

**Figure 1.**
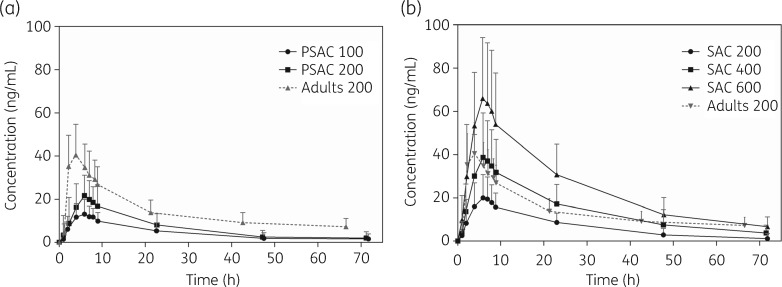
Mean concentration–time profiles of ascending doses of ivermectin in (a) PSAC and (b) SAC. Weight-dependent doses (μg/kg) are indicated as numbers in the keys. Results of adult volunteers are illustrated as a comparator.^18^

The mean concentration–time profiles of ascending doses of ivermectin administered to PSAC and SAC are illustrated in Figure 1, and PK parameters are summarized in Table 2. *C*_max_ increased with ascending doses, and median values of 15.5 and 24.4 ng/mL were obtained for PSAC treated with 100 and 200 μg/kg ivermectin, respectively, and 21.9, 40.7 and 66.1 ng/mL for SAC treated with 200, 400 and 600 μg/kg ivermectin, respectively. AUCs also correlated with dose, e.g. AUC_0__–__72_ increased from 169 to 369 ng×h/mL in PSAC and from 331 to 880 to 1636 ng×h/mL in SAC with ascending doses. The median *T*_max_ (5.92–6.80 h), *t*_1/2_ (16.3–19.1 h), MRT_INF_ (26.9–29.0 h), V/F (7.46–10.4 L/kg) and CL/F (5.68–8.58 L/h) were similar in the five treatment arms and thus independent of dose and age (2–12 years).

**Table 2.** PK and efficacy parameters of ivermectin in PSAC and SAC

Parameter	PSAC 100 μg/kg (*n = *39)	PSAC 200 μg/kg (*n = *41)	SAC 200 μg/kg (*n = *41)	SAC 400 μg/kg (*n = *39)	SAC 600 μg/kg (*n = *40)	Adults^18^ 200 μg/kg (*n = *11)
*C*_max_ (ng/mL)	15.5 (10.8–17.6)	24.4 (17.1–33.9)	21.9 (12.8–31.9)	40.7 (24.2–53.3)	66.1 (49.8–93.2)	40.1 (32.4–59.1)
*T*_max_ (h)	5.95 (4.03–6.73)	5.92 (4.94–7.03)	6.80 (5.88–7.41)	6.00 (5.82–7.05)	5.95 (5.82–7.57)	3.90 (2.37–5.87)
*t*_1/2_ (h)	17.3 (9.85–40.1)	16.3 (8.53–31.0)	18.1 (12.9–24.1)	19.1 (14.6–24.5)	18.8 (15.4–22.8)	32.3 (20.8–44.4)
AUC_0–72_ (ng×h/mL)	169 (103–438)	369 (225–669)	331 (199–634)	880 (680–1324)	1636 (1102–2140)	810 (608–1235)
AUC_INF_ (ng×h/mL)	310 (136–758)	500 (319–965)	662 (349–897)	1056 (775–1506)	1834 (1269–2436)	960 (782–1731)
AUMC_INF_ (ng ng×h^2^/mL)	5298 (3516–42 903)	11 403 (4787–49 061)	18 207 (6278–31 705)	31 644 (17 109–52 516)	57 526 (33 029–84 680)	53 488 (26 747–120 842)
MRT_INF_ (h)	29.0 (16.0–63.7)	26.9 (14.2–49.8)	28.5 (21.0–38.7)	27.8 (23.3–38.7)	28.7 (23.8–35.5)	48.7 (32.8–70.0)
CL/F (L/h)	6.17 (2.29–9.14)	5.68 (2.85–9.55)	7.75 (6.13–11.1)	8.58 (5.98–11.5)	7.40 (5.64–9.63)	11.5 (6.93–17.9)
CL/F (L/h/kg)	0.40 (0.14–0.67)	0.40 (0.21–0.63)	0.31 (0.23–0.49)	0.36 (0.28–0.59)	0.30 (0.25–0.46)	0.20 (0.14–0.26)
V/F (L/kg)	7.46 (5.68–10.8)	8.26 (6.73–12.6)	9.05 (6.98–10.5)	10.4 (8.42–13.0)	8.58 (7.22–12.2)	8.11 (6.07–12.4)
Cure rate (%)	10.8	20.5	2.50	2.70	12.8	NA
Egg reduction rate (%)	62.1 (33.5–79.5)	77.0 (55.8–88.6)	54.9 (35.4–69.5)	47.3 (17.4–68.0)	66.6 (44.1–81.0)	NA

PK parameters are presented as median (IQR), and efficacy data are presented as percentage (95% CI). PK data of adults are given as a reference. NA, not available.

AUC or *C*_max_ was computed as a function of absolute dose and weight or BMI, and the results, grouped by weight or BMI classes, are illustrated in the supplementary information. AUC as a function of weight increased dose proportionally in both age groups (Figure S1, available as Supplementary data at *JAC* Online). There was a statistically significant negative association of weight with AUC, even after adjustment for dose in SAC but not in PSAC. Similar results were found for BMI in PSAC, whereas the association of BMI with AUC vanished in SAC after adjustment for dose (Figure S2). Results for AUC and weight were similar to those for *C*_max_ and weight in PSAC. The association pattern was more complex in SAC. Here, *C*_max_ showed a faster than linear increase with ascending dose and the model including weight and an interaction between weight and dose provided a significantly better fit than the model without any weight terms or the model with weight but no interaction between dose and weight (Figure S3). The results for BMI and *C*_max_ resembled those for BMI and AUC in both SAC and PSAC (Figure S4).

**Figure 2.**
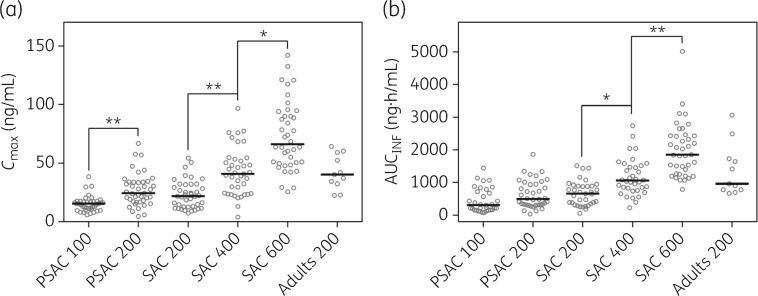
C_max_ and AUC_INF_ of ascending doses of ivermectin in PSAC and SAC. The median is illustrated as a black line. Weight-dependent doses (μg/kg) are indicated as numbers on the *x*-axes. Results of adult volunteers are illustrated as a comparator.^18^ *P < 0.016, **P < 0.009.

### PK–efficacy correlation for T. trichiura infections

Efficacy results are presented as cure rates and egg reduction rates (Table 2). Whereas correlations between egg reduction rates and AUC were negligible in SAC and PSAC, a significant positive association between ivermectin exposure measured by AUC_INF_ and cure rate was determined in SAC, with a change in the odds of cure rate by 8.3% with a 100 ng×h/mL increase in AUC_INF_ (95% CI 0.1%–17.2%, *P* = 0.047). No significant association was evaluated for PSAC.

## Discussion

Ivermectin is marketed for humans ≥15 kg (>5 years of age) to treat onchocerciasis, strongyloidiasis and lymphatic filariasis. Owing to the lack of an effective treatment against *T. trichiura* and other parasitic diseases, ivermectin appears to be a promising drug candidate with its broad antiparasitic activity. Despite its distribution to millions of children (>5 years of age), to our knowledge no paediatric PK characterization has yet been performed. For the first time, the disposition of ivermectin was evaluated in PSAC (2–5 years) and SAC (6–12 years) treated with ascending doses. Whereas drug exposure was similar in children of both age groups and increased with ascending dosage, AUCs were 2-fold lower in children than in adults when the same weight-dependent dose was administered. This finding can have major implications for the efficacy and safety for paediatric treatment of many diseases.

PK parameters, i.e. *T*_max_, *t*_1/2_, MRT_INF_, CL/F and V/F, were of similar value in all treatment arms, highlighting the comparability of ivermectin’s PK in the age range of 2–12 years. *C*_max_ and AUCs were similar in PSAC and SAC when the same weight-dependent dose was administered (200 μg/kg ivermectin) (Table 2) and increased with ascending doses. A statistically significant difference was observed between the treatment arms among SAC for *C*_max_ and AUC and among PSAC for *C*_max_ (Figure 2).

A positive association between AUC or *C*_max_ computed with the absolute dose (mg) and weight was observed in both age groups (Figures S1–S4). Interestingly, AUCs were significantly higher in PSAC of lower BMI. Ivermectin is a lipophilic drug and therefore accumulates in fat tissue. When ivermectin is administered to patients with higher BMI, it is likely that a larger proportion of ivermectin accumulates in fat tissue, leading to smaller amounts available in the blood and thus to lower AUC values. Helminth infections affect mostly children living in areas where undernutrition is a common public health problem. With more ivermectin being available in the blood in young children with a low BMI, systemic drug exposure is raised, possibly resulting in a different efficacy and safety profile; however, further studies are required to confirm this finding.

Despite moderate egg reduction rates, all doses administered to SAC and PSAC resulted in low cure rates against *T. trichiura* (<21%, Table 2) and in total only 7 SAC and 17 PSAC were cured.^19^ Nonetheless, logistic regression provided a statistically significant positive association between ivermectin exposure (AUC_INF_) and cure rate in SAC. To date, few PK studies have been conducted in participants infected with intestinal helminths, and it remains unknown whether AUC or *C*_max_ or solely intestinal concentrations are responsible for anthelminthic activity. A recent study in hookworm-infected children treated with tribendimidine did not identify a relationship between drug exposure and efficacy.^22^

Interestingly, the PK results of PSAC and SAC differ from our own findings in adults when treated with the current standard dose of 200 μg/kg.^18^ In more detail, *C*_max_ and AUCs are ∼2-fold higher in adults than in children (Figure 2). V/F generally depends on body compartments such as body water or fat content. No difference in V/F was identified between ivermectin-treated children (2–12 years) and adults, indicating a similar ratio of body compartments (Table 2). CL/F is similar in all paediatric treatment arms but significantly higher than CL/F of adults when the values are adjusted to body weight (Table 2 and Figure S5). Other PK parameters (*t*_1/2_, MRT_INF_ and CL/F) were similar in all treatment arms of the two paediatric populations but are only approximately half of the values observed in adults, and *T*_max_ is lower in adults than in children (3.90 h versus 5.92–6.80 h, Table 2). Of note, our PK studies with either children or adults were performed in the same setting in southern Côte d’Ivoire, followed the same protocol, including DBS sampling, and all participants harboured a *T. trichiura* infection. Thus, the difference in these results cannot be explained by study design, infection, procedures or ethnicity of volunteers. However, only limited information on the PK of ivermectin in humans is available. Yet, it is widely known that the function and characteristics of the gastrointestinal tract (e.g. pH, motility and transit time), hepatic and renal function, and metabolic processes alter with age and thus can influence the PK of a drug.^23^^,^^24^ Indeed, age-dependent variation in the PK of ivermectin has been reported in different animal species, in which ivermectin was studied more intensively.^25^ Intestinal motility is decreased in children, causing impaired transit time (3–7.5 h in children; 3–4 h in adults).^26^ This physiological difference might explain the prolonged uptake of ivermectin leading to a higher *T*_max_ in children. Moreover, intestinal motility is responsible for drug–mucosa interaction. If this process is impaired, a lower amount of ivermectin will be absorbed, causing lower *C*_max_ and AUC. This might be supported by the lower blood supply by the superior mesenteric artery to the intestine in children than in adults (377 and 517 mL/min, respectively).^26^ Ivermectin is primarily metabolized in the liver, and hepatic damage caused by viral hepatitis or alcoholism in adults could be an additional explanation of different ivermectin levels.^27^ It is worth highlighting that most studies evaluating physiological development with age are based on Western standardized body values and do not consider ethnic differences or the influence of infections and malnutrition.

It has been repeatedly expressed that children are not small adults and drug dosages cannot be simply extrapolated from adults to children by adjusting for the body weight. The WHO highlighted the need for licensed paediatric drugs as still millions of children suffer owing to untreated diseases, but barriers for PK trials in children remain high owing to ethical and technical challenges. PK modelling and simulations based on data derived from adults might aid in providing paediatric treatment recommendations. Recently simulated PK parameters of ivermectin of healthy adults resemble our PK parameters of adults.^18^^,^^28^ However, the simulated parameters of children based on adults’ data differ from our results with, for example, predicted approximately double *C*_max_ and approximately half *T*_max_.^28^ Therefore, the present study highlights once more that PK clinical trials including paediatric studies are essential to understanding drugs, especially when undernutrition and intestinal infections are common.

In summary, a phase II clinical trial was performed in two paediatric populations (PSAC and SAC) infected with *T.trichiura*, and PK parameters of ascending doses of ivermectin were evaluated in micro-blood DBS samples. AUC and *C*_max_ increased with ascending doses, and *T*_max_, *t*_1/2_, MRT_INF_, CL/F and V/F were dose and age independent. Malnutrition or undernutrition might influence the AUC of ivermectin in small children. Ivermectin shows a lower exposure profile in children than in adults, highlighting the need to study drug dosing carefully, in particular given the great interest in applying this drug for novel indications.

## Supplementary Material

dkz083_Supplementary_Data
