# Supplementary material for: Pharmacokinetics of ascending doses of ivermectin in Trichuris trichiura-infected children aged 2–12 years
Source: J Antimicrob Chemother. 2019 Mar 11;74(6):1642–7. doi: 10.1093/jac/dkz083 (PMC6524481; doi:10.1093/jac/dkz083)
Supplement: dkz083_Supplementary_Data [file dkz083_supplementary_data.doc]

**Supplementary data**

A

B

**
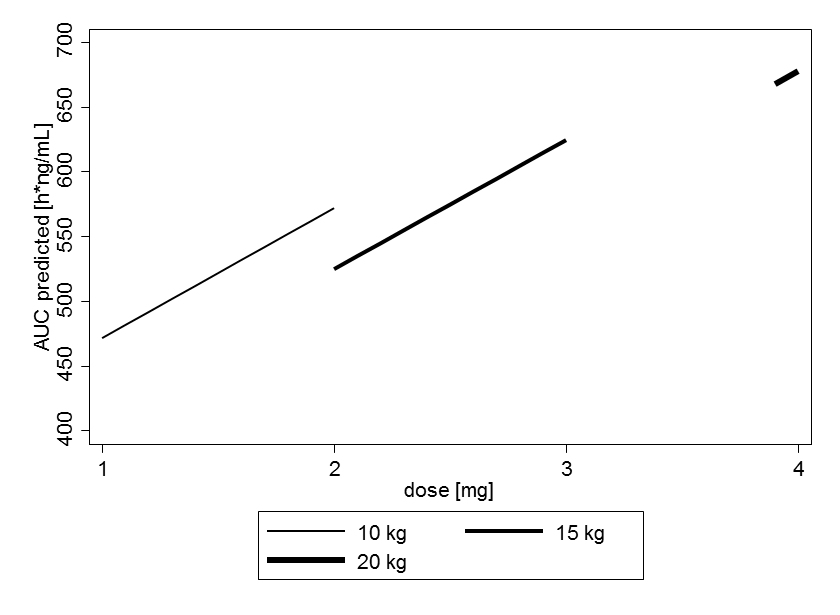

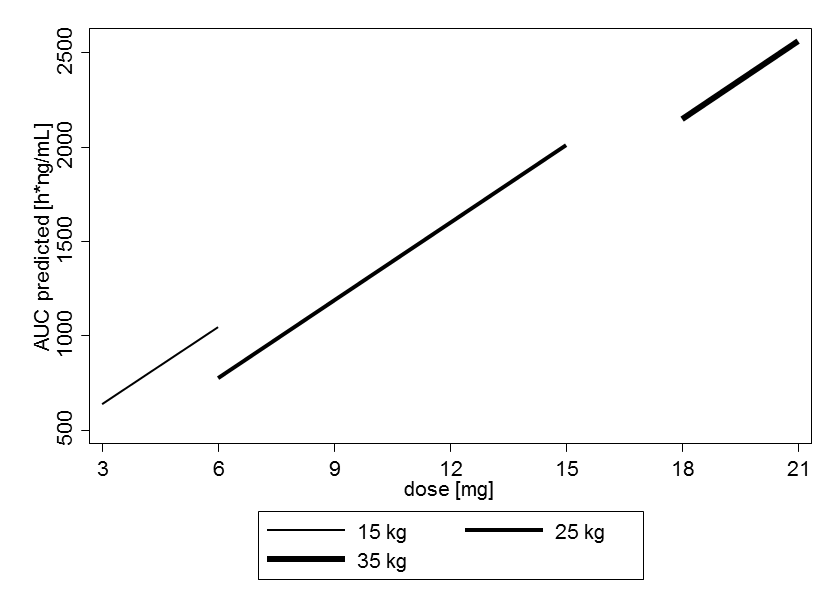
**

**Figure S1.** AUC_INF_ as a function of dose and weight of (A) preschool-aged children (PSAC) and (B) school-aged children (SAC). The dose-responsive slope decreased significantly with increasing weight in SAC (p < 0.05). Curves were obtained from a model of the form AUC = b_0_ + b_1_ × weight + (b_2_ + b_3_ × weight) × dose^exp(b4)^.The Akaike information criterion suggested that b_3_ = 0 and b_4_ = 0.

A

B


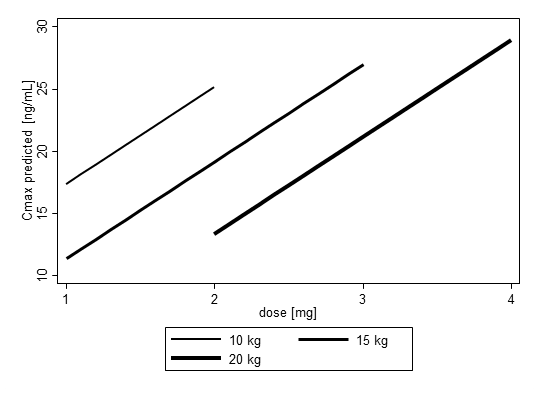
**
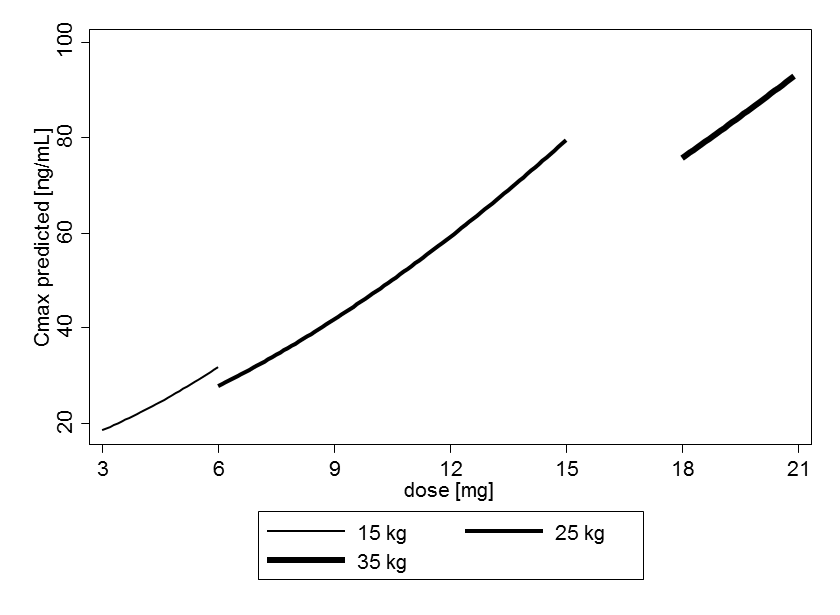
**

**Figure S2.** C_max_ as a function of dose and weight of (A) PSAC and (B) SAC. The dose-responsive slope decreased significantly with increasing weight in PSAC (p < 0.05). Curves were obtained from a model of the form C_max_ = b_0_ + b_1_ × weight + (b_2_ + b_3_ × weight) × dose^exp(b4)^. The Akaike information criterion suggested that b_3_ = 0 and b_4_ = 0 in PSAC and b_3_, b_4_ > 0 in SAC.

A

B


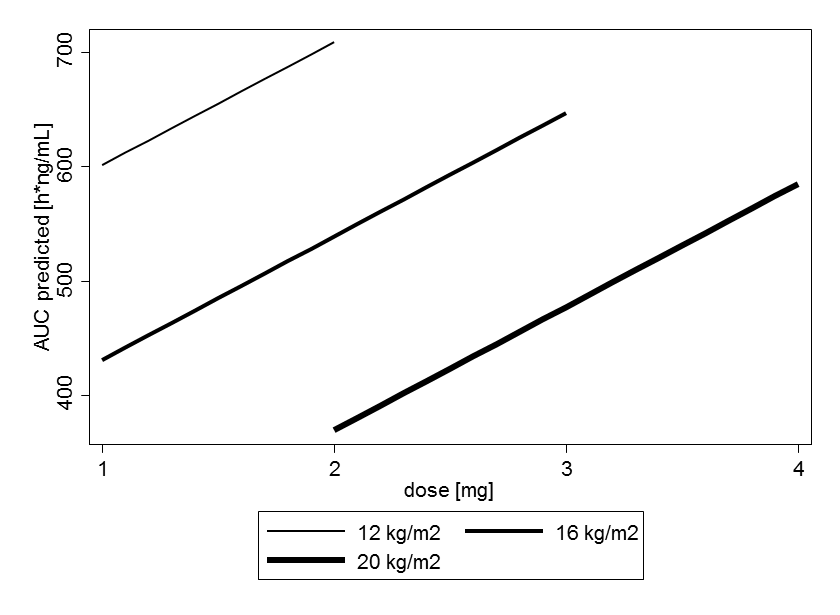

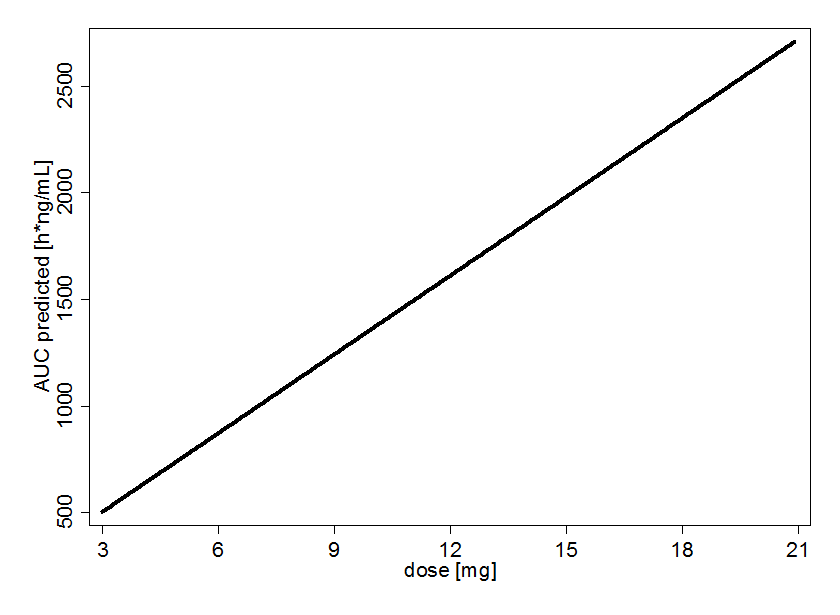


**Figure S3.** AUC_INF_ as a function of dose and BMI of (A) PSAC and (B) SAC. The dose-responsive slope decreased with increasing BMI in PSAC (p < 0.06). Curves were obtained from a model of the form AUC = b_0_ + b_1_ × BMI + (b_2_ + b_3_ × BMI) × dose^exp(b4)^ . The Akaike information criterion suggested that b_3_ = 0 and b_4_ = 0. Moreover, the influence of BMI was negligible in SAC.

A

B


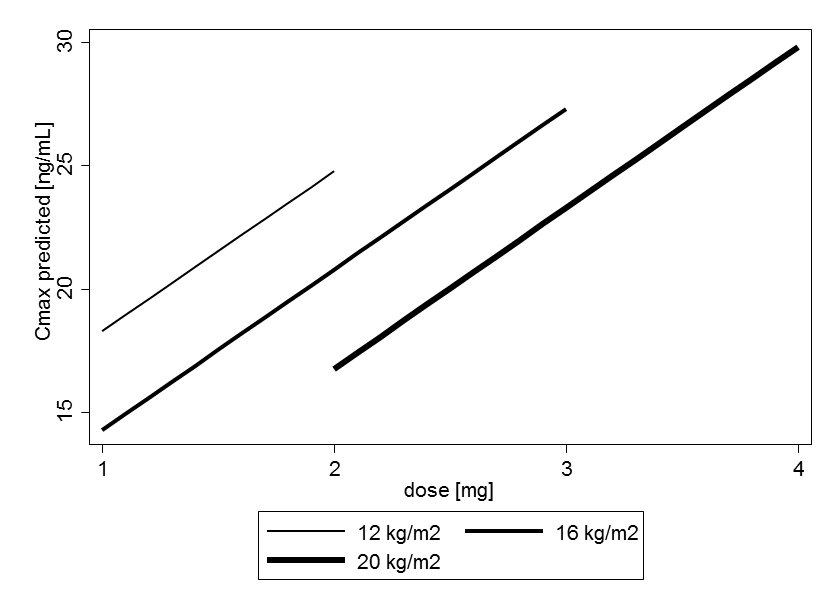

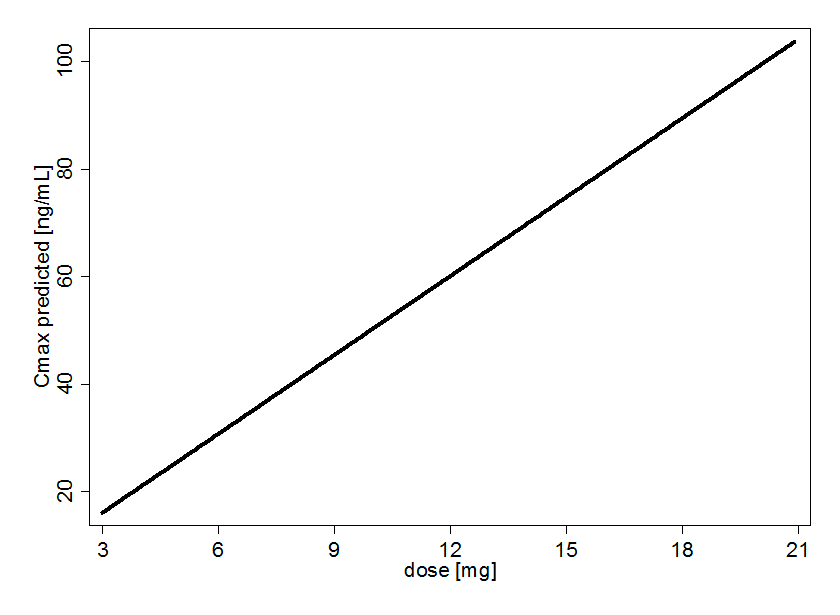


**Figure S4.** C_max_ as a function of dose and BMI of (A) PSAC and (B) SAC. Curves were obtained from a model of the form AUC = b_0_ + b_1_ × BMI + (b_2_ + b_3_ × BMI) × dose^exp(b4)^. The Akaike information criterion suggested that b_3_ = 0 and b_4_ = 0. Moreover, the influence of BMI was negligible in SAC.


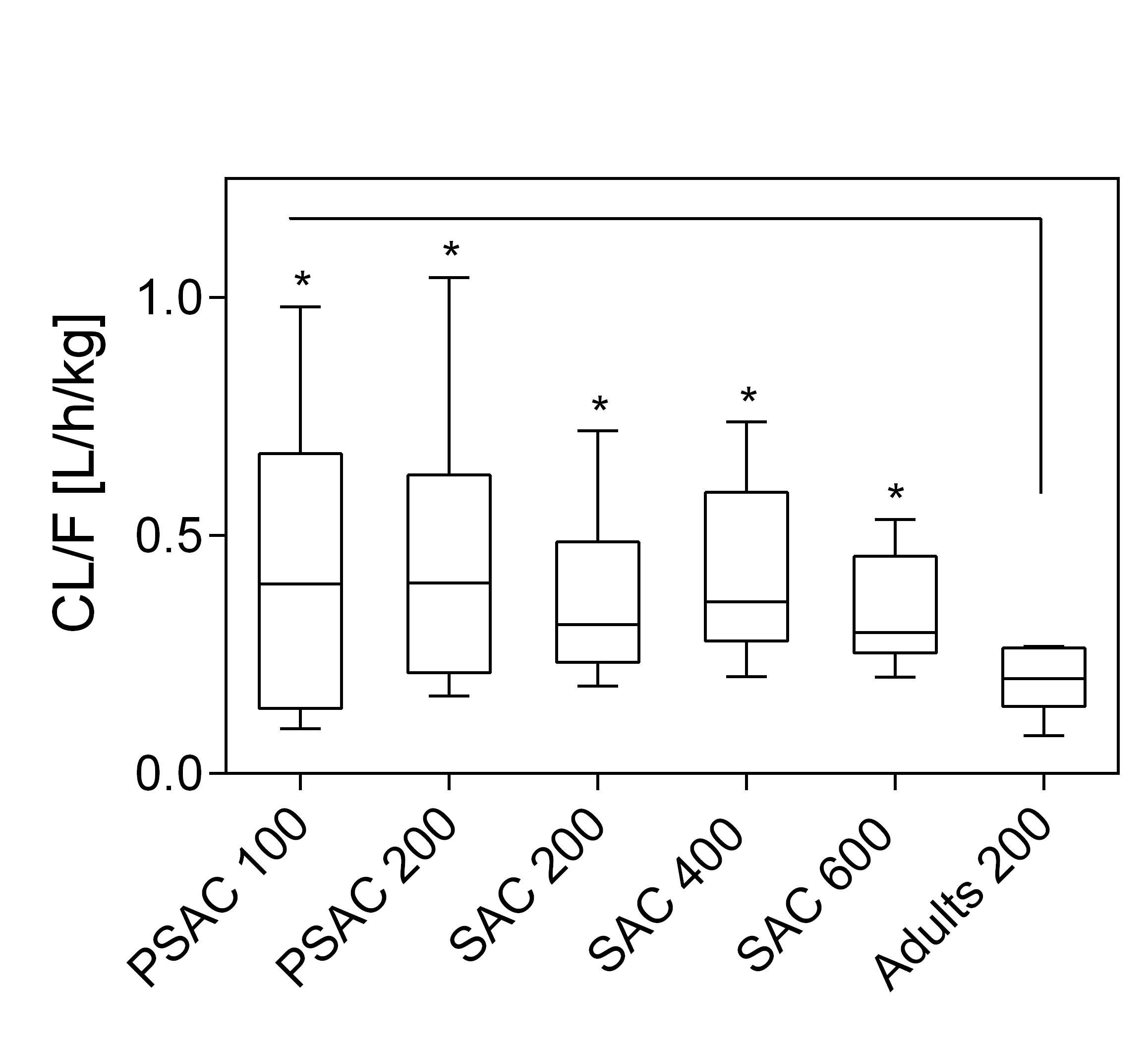


Figure S5. CL/F of ascending doses of ivermectin in PSAC and SAC. Median and IQR are illustrated with 10–90 percentile. Weight-dependent doses (µg/kg) are indicated as numbers on the x-axes. Results of adult volunteers are illustrated as a comparator.^18^ **p* < 0.017
